# Analysis of Chromosome Associations during Early Meiosis in Wheat Lines Carrying Chromosome Introgressions from *Agropyron cristatum*

**DOI:** 10.3390/plants10112292

**Published:** 2021-10-25

**Authors:** Pilar Prieto, Carmen Palomino, Zuny Cifuentes, Adoración Cabrera

**Affiliations:** 1 Plant Breeding Department, Institute for Sustainable Agriculture, Agencia Estatal Consejo Superior de Investigaciones Científicas (CSIC), Alameda del Obispo s/n, Apartado 4048, 14080 Córdoba, Spain; zmcifuentes@ias.csic.es; 2 Genetics Department, ETSIAM, Campus de Rabanales, Universidad de Córdoba, CeiA3, 14071 Córdoba, Spain; ge1pasam@uco.es (C.P.); ge1cabca@uco.es (A.C.)

**Keywords:** *Agropyron cristatum*, wheat, chromosome pairing, *Ph1*, introgression

## Abstract

Crested wheatgrass (*Agropyron cristatum* L. Gaertn., genome P), included in the Triticeae tribe (family Poaceae), is one of the most important grasses in temperate regions. It has been valued as a donor of important agronomic traits for wheat improvement, including tolerance to cold, drought, and high salinity, as well as resistance to leaf rust, stripe rust, and powdery mildew. For successful incorporation of beneficial alleles into wheat, it is essential that recombination between wheat and *A. cristatum* chromosomes occurs. In this work, we analysed chromosome associations during meiosis in wheat lines carrying chromosome introgressions from *A. cristatum* chromosomes 5P and 6P in the presence and absence of *Ph1* locus using fluorescence *in situ* hybridisation. The results showed that the *Ph1* locus does not affect chromosome associations between *A. cristatum* and wheat chromosomes because there were no interspecific chromosome associations; therefore, no recombination between chromosomes from wheat and *Agropyron* were observed in the absence of the *Ph1* locus. The 5P and 6P *A. cristatum* chromosomes do not have a suppressor effect on the *Ph1* locus. Wheat univalents in metaphase I suggest that *Agropyron* chromosomes might carry genes having a role in wheat homologous chromosome associations. Putative effect of the *Agropyron* genes on wheat chromosome associations does not interact with the *Ph1* locus.

## 1. Introduction

Crested wheatgrass (*Agropyron cristatum* L. Gaertn.) is a Triticeae species (family Poaceae) and has diploid (2n = 2x = 14), tetraploid (2n = 4x = 28), and hexaploid (2n = 6x = 42) forms, all based on the basic genome P [1]. *A. cristatum* is a perennial species of economic importance that is widely cultivated in North America as an excellent source of forage, and included in the diets of beef and dairy cattle worldwide [2]. Moreover, this species shows tolerance to cold, drought, and high salinity, and is one of the most important grasses in temperate regions [3,4]. It has also been valued for stabilization of heavy metal-contaminated soils [5,6] and watershed management [7]. In a wheat breeding framework, *A. cristatum* is an appreciated donor of important agronomic traits, such as resistance to leaf rust [8], resistance to powdery mildew [9,10], resistance to stripe rust [11], high salt-tolerance [12], drought stress tolerance [13], and enhanced-grain number per spike and spike length [14]. Hybrids and fertile amphiploids between *Triticum* and *A. cristatum* have been successfully obtained [15,16,17,18] with the aim to develop wheat-*A. cristatum* introgressions [8,14,19].

Recombination between cultivated and alien chromosomes is essential for incorporating beneficial alleles into wheat from their wild relatives. In hexaploid wheat (*Triticum aestivum* L., 2n = 6x = 42, genomes AABBDD), chromosome pairing and recombination is mainly controlled by pairing the homoeologous (*Ph1b*) gene located on the long arm of chromosome 5B. This gene is responsible for the diploidization system that allows regular meiosis in polyploid wheat [20,21]. Elimination, manipulation, or 5B chromosome activity suppression have been utilized to induce homoeologous chromosome pairing in wheat, for example, the use of 5B-deficient stocks [22]. In addition, the homoeologous pairing suppressor allele, *Ph2*, located on short arm of the 3D chromosome of wheat, has also been used [23], although the recessive mutant of the homoeologous pairing suppressor *Ph1b* is probably widely used and the most effective method for the induction of homoeologous pairing thus far [24]. A deletion mutant in the *Ph1b* gene has been the determinant in the development of introgressions between alien and wheat genomes by pairing between homoeologous chromosomes [24].

Other methods that promote homoeologous pairing are based on the development of genetic crosses between wheat and *Ae. Speltoides,* which suppress the action of the *Ph1* gene due to the presence of the *Ph1* suppressor genes *Su1-Ph1* and *Su2-Ph1* [25]. Epistatic genes to *Ph1* promoting homoeologous pairing and recombination have also been reported in hybrids of wheat with *Aegilops mutica* Boiss. [26]. Individual alien chromosomes added to wheat have also been shown to enhance homoeologous pairing by suppression the activity of *Ph* genes in wheat, such as chromosome 5U of *Aegilops umbellulata* Zhuk. [27], chromosome 5E and 6E of *Elytrigia elongata* (Host) Nevski. [28], and chromosome 5Mg of *Ae. geniculata* Roth [29].

Although some information on chromosome pairing in *Agropyron* and wheat hybrids and amphiploids is available [17,30], and a possible interaction of genes from *Agropyron* with the *Ph* system in wheat has been postulated [31], little is known about the “true” pairing frequency between *A. cristatum* and wheat chromosomes. Thus, the objective of this work was to analyse chromosome associations during meiosis in wheat lines carrying chromosome introgressions from *A. cristatum* in the presence and in the absence of the *Ph1* locus in the wheat genetic background.

## 2. Results

### 2.1. Characterization of 5P and 6P A. cristatum Introgression Lines in Wheat in the Presence and in the Absence of the Ph1 Locus

**Figure 1:**
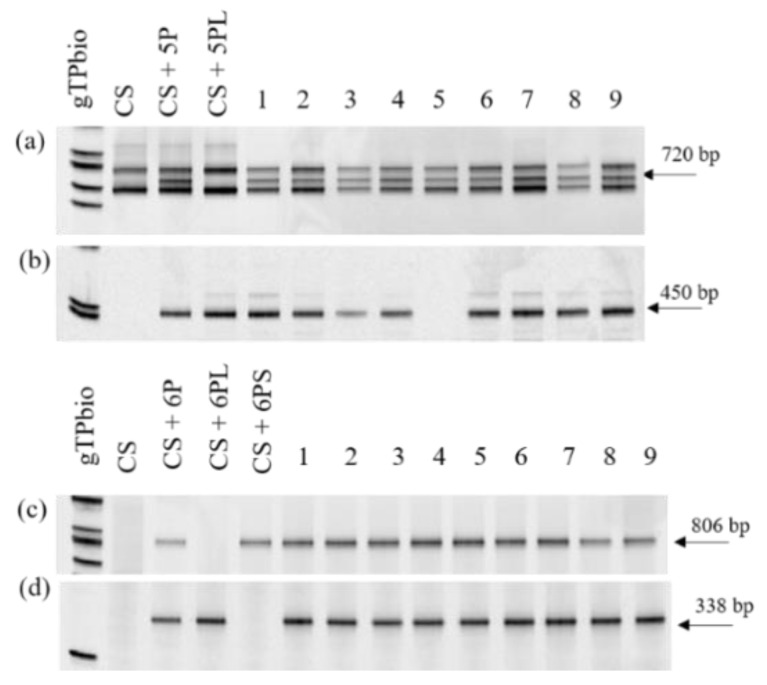
PCR amplification profiles used for the identification of chromosome 5P and 6P in the wheat addition lines. (**a**) COS108 and (**b**) COS150 mapped on the short and the long arm of chromosome 5P, respectively. (**c**) COS440 and (**d**) COS507 mapped on short and the long arm of chromosome 6P, respectively. All lines carried a complete chromosome, except for line 5 in in (**a**,**b)** which was ditelosomic for the 5PS chromosome arm.

**Figure 2:**
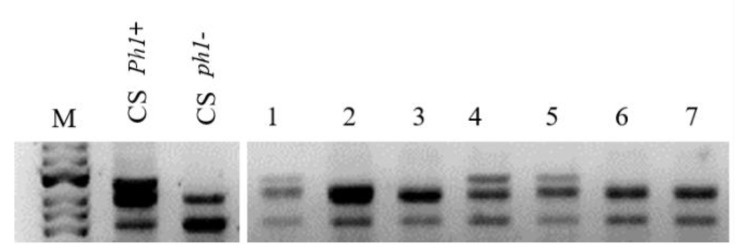
Genotypic assays for the presence of *Ph1*. The absence of *Ph1* is marked by the ABC920 SCAR marker (individuals 2, 3, 6, and 7). M: size marker; CS: *Triticum aestivum* cv Chinese Spring; *Ph1*+: wild type wheat CS; and *Ph1*−: the parental *Ph1* mutant.

Addition lines for chromosomes 5P and 6P in the *Ph1* genetic background were obtained in the F_3_ progeny from crosses between *A. cristatum* additions for chromosomes 5P and 6P in wheat CS with the CS *Ph1* mutant, respectively [32]. To detect the presence of 5P or 6P chromosomes in the presence and in the absence of the *Ph1* locus, we used specific COS molecular markers for the short and the long arms of these two chromosomes (Figure 1). Zygosity at the *Ph1* locus was predicted using *Ph1* diagnostic ABC920 SCAR marker (Figure 2).

**Figure 3:**
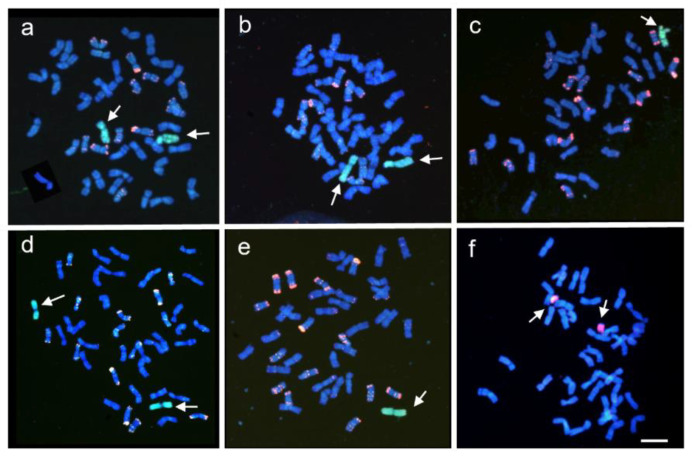
FISH to mitotic metaphase chromosome spreads. (**a**) Disomic CS+5P, *Ph1*; (**b**) Disomic CS+5P, *ph1ph1*; (**c**) Monosomic CS+5P, *ph1ph1*; (**d**) Disomic CS+6P, *Ph1*; (**e**) Monosomic CS+6P, *ph1ph1*; and (**f**) Ditelosomic CS+5P, *Ph1*. In (**a**–**e**), double FISH signals with the pAs1 probe (red) and *A. cristatum* genomic DNA (green) as probes; In (**f**), FISH signals with *A. cristatum* genomic DNA (red). Scale bar represents 10 µm.

Based on the analysis using the *Ph1* diagnostic marker, we identified homozygous plants for *Ph1* and positives for both COS108 and COS150 markers located on the short and the long arm of chromosome 5P, respectively (Figure 1a,b). Fluorescence *in situ* hybridisation (FISH) analysis demonstrated that these plants were disomic (Figure 3b) or monosomic (Figure 3c) for chromosome 5P in the *Ph1* genetic background, respectively. In addition, we identified one ditelosomic *A. cristatum* introgression line for chromosome 5PS in the *Ph1* genetic background, which only amplified the fragment for the COS108 marker located on the short arm of chromosome 5P, but not the one for the COS150 marker located on chromosome 5PL. FISH analyses demonstrated that this plant was ditelosomic for the 5PS arm (Figure 3f). The 5PS ditelosomic plant was selfed and the descendance was also evaluated using COS molecular markers. All plants obtained were ditelosomic for the 5PS chromosome arm, indicating that the 5PS arm was stably inherited to the descendance. All plants were fertile and vigorous. Similarly, positive plants for both COS440 and COS507, specific for the short and the long arm of chromosome 6P, respectively, were detected in the absence of the *Ph1* locus (Figure 1a,d) indicating that these plants carried the complete 6P chromosome in the *Ph1* mutant background. FISH analysis revealed that these plants were monosomic for chromosome 6P (Figure 3e) and no plant carrying two copies of chromosome 6P in the absence of the *Ph1* locus was obtained.

### 2.2. Early Meiosis Analysis of 5P and 6P A. cristatum Introgression Lines in Wheat in the Presence and in the Absence of the Ph1 Locus

With the aim of assessing a putative effect of the *A. cristatum* genome on chromosome pairing and recombination during meiosis in wheat, chromosome associations were analysed in pollen mother cells (PMCs) during early meiosis by genomic *in situ* hybridisation (GISH) in plants carrying one or two copies of chromosomes 5P and 6P from *A. cristatum*, respectively, in the presence of the *Ph1* locus. Observations on chromosome associations in the presence of the *Ph1* locus were also compared to equivalent 5P and 6P addition lines in wheat in the absence of the *Ph1* locus. We unfortunately did not obtain any plant carrying two copies of chromosome 6P in the absence of the *Ph1* locus. Thus, we were only able to compare the effect of the *Ph1* locus in disomic 5P plants.

**Figure 4:**
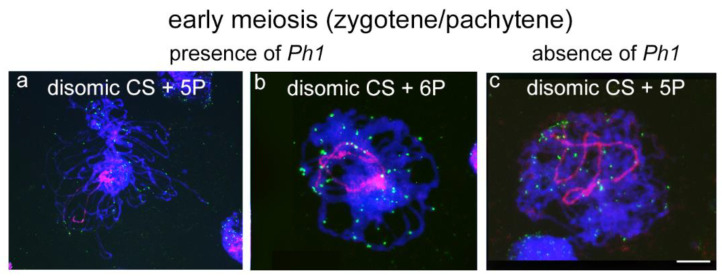
FISH to chromosome spreads in early meiosis stages (zygotene–pachytene) in wheat lines carrying chromosomes 5P or 6P from *A. cristatum*, both in the presence and in the absence of the *Ph1* locus. Simultaneous visualization of telomeres (green) and *A. cristatum* chromosomes (red). Wheat DNA was counterstained with DAPI (blue). *Agropyron cristatum* homologous chromosomes were visualized associated in pairs in all the panels. (**a**) Disomic CS+5P, *Ph1Ph1*; (**b**) Disomic CS+6P, *Ph1Ph1*; and (**c**) Disomic CS+5P *ph1ph1*. The scale bar represents 10 µm.

Experiments were developed in more than one hundred PMCs of each genomic combination in both early prophase I of meiosis (zygotene, pachytene) and metaphase I, and both in the presence and in the absence of the *Ph1* locus. When two copies of 5P or 6P chromosomes were present in the presence of the *Ph1* locus, both *A. cristatum* chromosomes were observed in proximity in the nucleus in early prophase (data not shown). As meiosis progressed, GISH experiments in zygotene–pachytene stages showed homologous 5P *A. cristatum* chromosomes always fully associated in pairs along the whole chromosome in all the cells analysed (Figure 4a). Similarly, homologous 6P *A. cristatum* chromosomes were also observed associated at these early meiotic stages in all the cells visualized (Figure 4b). These observations suggested that *A. cristatum* chromosomes associated correctly in pairs in the wheat background in early meiosis, and no genetic interaction between wheat and *A. cristatum* chromosomes seemed to be allowed in the presence of the *Ph1* locus at these stages. Similarly, experiments in the absence of the *Ph1* locus showed that homologous 5P *A. cristatum* chromosomes were also associated in pairs in early pachytene in all the cells analysed (Figure 4c) suggesting that chromosome interactions between wheat and *A. cristatum* chromosomes were not promoted in early meiosis in the absence of the *Ph1* locus.

### 2.3. Recombination between A. cristatum and Wheat Chromomomes Does Not Occur Either in the Presence or in the Absence of the Ph1 Locus

**Figure 5:**
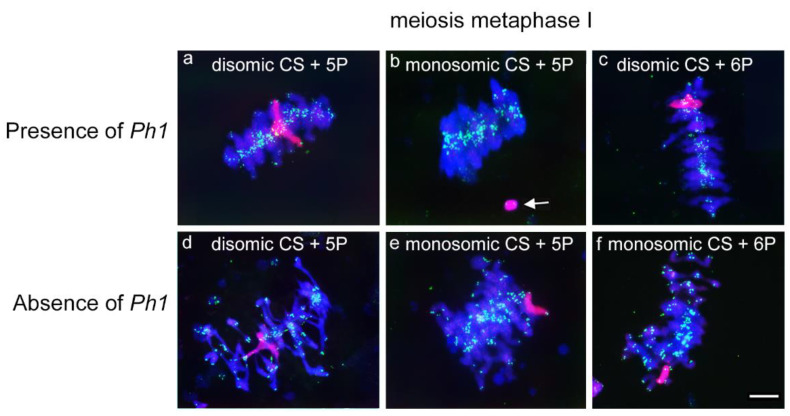
FISH analysis of chromosome associations in metaphase I in wheat lines carrying chromosomes 5P or 6P from *A. cristatum*, both in the presence and in the absence of the *Ph1* locus. Homologous 5P and 6P chromosomes (red) are visualized associated in disomic lines, independent of the presence of the *Ph1* locus. Telomeres and wheat chromosomes are visualized in green and blue, respectively. (**a**) Disomic CS+5P, *Ph1Ph1*; (**b**) Monosomic CS+5P, *Ph1Ph1;* (**c**) Disomic CS+6P, *Ph1Ph1;* (**d**) Disomic CS+5P, *ph1ph1;* (**e**) Monosomic CS+5P, *ph1ph1;* and *(***f**) Monosomic CS+6P, *ph1ph1.* The scale bar is 10 µm.

Once we observed full chromosome associations between homologous *A. cristatum* chromosomes for both 5P and 6P during early meiosis in the wheat background either in the presence of in the absence of the *Ph1* locus, we also analysed chromosome behaviour of both 5P and 6P *A. cristatum* chromosomes during metaphase I of meiosis, although interspecific recombination events were not expected, due to the lack of interspecific chromosome pairing earlier in meiosis. Thus, wheat chromosomes were observed associated correctly in bivalents and oriented by centromeres properly at meiosis metaphase I in most of the PMCs analysed from 5P and 6P disomic additions in wheat lines carrying the *Ph1* locus (Figure 5). In addition, both homologous 5P and 6P chromosomes were also visualized correctly associated in pairs in the presence of the *Ph1* (Figure 5a,c). Moreover, in the 5P monosomic addition line, the *A. cristatum* chromosome remained always unassociated in all the cells analysed in the presence of the *Ph1* locus (Figure 5b). Our observations clearly suggested that recombination did not occur in meiosis between these *A. cristatum* and wheat chromosomes in the presence of the *Ph1* locus, even when only one copy of *A. cristatum* is present. Thus, 5P *Agropyron* chromosomes were never observed associated with wheat chromosomes by chiasmata during metaphase I in the 5P *A. cristatum* monosomic addition line, always remaining as univalent (Figure 5b) and suggesting other requirements for chromosome associations and recombination.

We also focused on studying whether chromosome associations, and therefore overcrossing and recombination events, between *A. cristatum* and wheat chromosomes could be allowed in the absence of the *Ph1* locus. Thus, we analysed chromosome associations in PMCs in metaphase I samples from disomic and monosomic *A. cristatum* addition lines in wheat in the absence of the *Ph1* locus. As expected, homologous 5P *A. cristatum* chromosomes that associated previously in early meiosis (Figure 4c) remained associated in metaphase I in disomic addition lines in wheat in the absence of the *Ph1* (Figure 5d). In the absence of homologues, chromosomes 5P and 6P from *A. cristatum* also always remained as univalents during metaphase I in GISH experiments in 5P and 6P monosomic addition lines in the absence of the *Ph1* locus in all the cells analysed (Figure 5e,f). These results showed that interspecific chromosome associations between *A. cristatum* and wheat chromosomes are not promoted in the absence of the *Ph1* locus, suggesting that chromosome associations might also depend on other elements, such as genome homology, as *A. cristatum* and wheat species are phylogenetically distant. Furthermore, the absence of homologous chromosomes did not contribute to increasing chromosome associations between *A. cristatum* and wheat chromosomes, which did not occur either in the presence or in the absence of the *Ph1* locus. In summary, our observations suggested that the *Ph1* locus does not affect chromosome associations between *A. cristatum* and wheat chromosomes because no interspecific chromosome associations and recombination between wheat and *Agropyron* chromosomes were found in either in the presence or in the absence of the *Ph1* locus. Our results also suggest that genes carried in 5P and 6P *A. cristatum* chromosomes do not have a suppressor effect on the *Ph1* locus, as the observations were equivalent both in the presence and in the absence of the *Ph1* locus.

### 2.4. The Presence of Both 5P and 6P A. cristatum Chromosomes Affects Chromosome Associations and Recombination between Homologous Wheat Chromosomes

**Figure 6:**
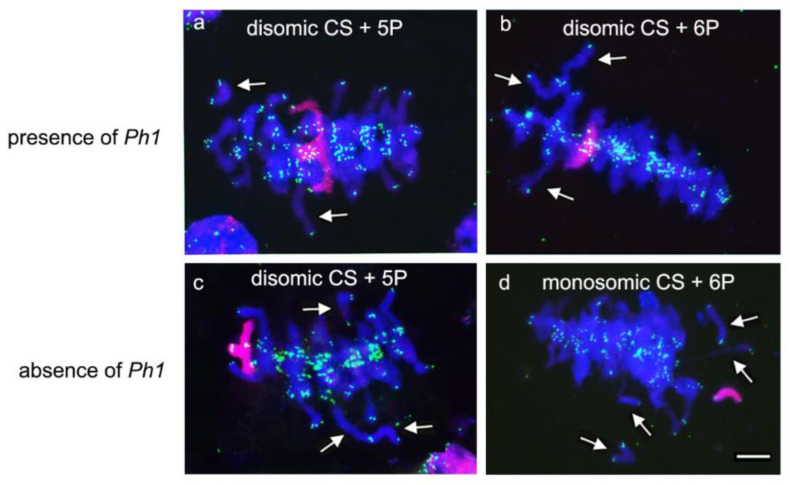
FISH analysis in metaphase I chromosome spreads from wheat lines carrying 5P or 6P *A. cristatum* chromosomes, both in the presence and in the absence of the *Ph1* locus. Representative metaphase I cells showing wheat univalents (arrowed) both in the presence and in the absence of the *Ph1* locus. Homologous 5P and 6P chromosomes (red) are visualized associated in disomic lines, independent of the presence of the *Ph1* locus. Telomeres are shown in green and wheat chromosomes in blue. (**a**) Disomic CS+5P, *Ph1Ph1;* (**b**) Disomic CS+6P, *Ph1Ph1;* (**c**) Disomic CS+5P, *ph1ph1;* (**d**) Monosomic CS+6P, *ph1ph1.* Scale bar is 10 µm.

Alterations in chromosome pairing between wheat chromosomes were found during GISH experiments developed in meiosis in PMCs from wheat lines carrying both the 5P and the 6P *A. cristatum* chromosomes. In fact, a high number of cells in metaphase I showed wheat chromosomes remaining as univalents both in the presence and in the absence of the *Ph1* locus when both the 5P and 6P *A. cristatum* chromosomes were present in the wheat background (Figure 6). Cells carrying one, two, or three unassociated wheat chromosomes were scored in metaphase I cells in both cases, in the presence and in the absence of the *Ph1* locus (Table 1). These results suggested that the *Agropyron* chromosomes carried genes having a role in homologous chromosome associations as correct wheat homologous pairing was disrupted in the presence of both 5P and 6P *Agropyron* chromosomes. Our observations also suggested that the putative effect of the *Agropyron* genes on homologous chromosome associations does not have any interaction with the *Ph1* locus because the observations of wheat univalents were equivalent both in the presence and in the absence of the *Ph1* locus.

## 3. Discussion

The P genome from *A. cristatum* contains a high genetic diversity, which has been demonstrated by cytological, molecular, and morphological data [33,34]. The *A. cristatum* genome has also been considered a valuable source of genes for pest resistance and abiotic stresses in the framework of wide hybridization programs to improve cereal crops [2,35]. For example, hybrids between *Triticum* and *A. cristatum* have been obtained to introduce traits from *A. cristatum* into *Triticum* [15]. Both durum and common wheat *A. cristatum* introgression lines have also been developed [8,14,18,19] and they could be a useful source of agronomic traits, such as disease resistance [9,10,11], abiotic stresses [12,13], thousand grain weight [14], and grain quality [36].

Understanding the genomic relationship between the donor and the recipient species is essential for successful introgression of alien chromatin into the wheat genetic background and therefore, for an effective utilization of the large *Agropyron* gene reservoir for wheat breeding purposes. However, in a plant breeding context, the development of genetic introgressions from species included in the wheat tertiary gene pool, such as *A. cristatum*, is much more difficult to achieve than from those species belonging to both primary and secondary genetic pools, because they are phylogenetically more distantly related. *A. cristatum* includes diploid and polyploid forms, all based on the basic genome P [1]. Nevertheless, genetic studies have indicated that synteny is conserved between wheat and the P genome. For example, a high transferability of COS molecular markers between *A. cristatum* and wheat has been reported [9,37]. Similar results were obtained using FISH of tandem repeats and wheat single-gene probes [38,39]. Genetic linking between *A. cristatum* and wheat genomes has also been revealed by both sequencing the transcriptome of a tetraploid *A. cristatum* [40] and genetic mapping using a wheat 660K SNP array [41]. Particularly for homologous groups 5 and 6, comparative mapping using a set of COS molecular markers showed that most of them were located on the short or long arms of wheat chromosome group 5 and 6 and were assigned to corresponding short or long arms of *A. cristatum* 5P and 6P chromosomes, respectively, indicating both homology between chromosomes 5P and 6P and wheat group 5 and 6, respectively [37]. In addition, synteny between *A. cristatum* and wheat homologous group 5 has also been demonstrated by mapping agronomically important genes, such as the grain hardness (*Ha*) and the vernalization (*VRN-1*) loci on the short and on the long arms of chromosome 5P, respectively [36,42], showing that these loci are collinear with those located on the short and the long arms, respectively, of chromosomes 5A, 5B and 5D in wheat. Together, these results indicated that a conservation of genes exists between wheat and *A. cristatum* chromosomes. However, in this work, no homoeologous chromosome pairing among wheat and *A. cristatum* has been found either in the presence or in the absence of the *Ph1* locus. The results obtained suggest that the *Ph1* locus does not affect chromosome associations between *A. cristatum* and wheat genomes because no interspecific chromosome associations and recombination between wheat and *Agropyron* chromosomes were allowed in the absence of the *Ph1* locus. Our observations also suggested that the lack of interspecific chromosome pairing, even in the absence of the *Ph1* locus, might be due to the divergence of repetitive sequences between wheat and *A. cristatum* homoelogous group 5 and 6. In fact, specific sequences located on the distal chromosome regions (subtelomeres) might be hampering interspecific chromosome associations between these *Agropyron* and their homoeologous wheat chromosomes [43,44]. Nevertheless, translocations between wheat and *Agropyron* species have been previously achieved for transferring important traits, such as leaf and stem rust resistance genes from *A. elongatum* [45,46], wheat streak mosaic [47], and stem rust resistance genes from *A. intermedium* [48] to wheat background. Wheat *A. cristatum* Robertsonian translocations have also been obtained in the absence of the *Ph1* locus [32]. Actually, Robertsonian translocations are the most common chromosome rearrangements found between wheat and related species, both in the presence and in the absence of the *Ph1* locus [49,50], although other interstitial recombination events between wheat chromosomes and those from other relatives have been promoted in the *Ph1* mutant background [51].

Previous meiotic analyses in hybrids between wheat and the *Agropyron* species have shown high levels of chromosome pairing. For example, multivalent configurations were observed in hybrids between *T. aestivum* and tetraploid *A. cristatum* [30], and in the hybrid between *T. aestivum* and tetraploid *A. fragile,* leading to the conclusion that *A. fragile* has a genetic system that modified the *Ph1* gene activity [31]. Thus, interactions of genes from *Agropyron* with the *Ph1* system in wheat were considered the most plausible explanation for those multivalent configurations [31]. However, GISH analysis in the *T. tauschii*-*A. cristatum* (DDPP) amphiploid [17] revealed later that the high pairing observed was ascribable between different P chromosomes or different wheat chromosomes, but not between *Agropyron* and wheat chromosomes. The observed configurations between P chromosomes could be explained because of the segmental allopolyploid nature of tetraploid *A. cristatum*. Our results using GISH analysis support these observations as we did not find chromosome associations between *A. cristatum* and wheat chromosomes, either in the presence or in the absence of the *Ph1* locus. In addition, we observed wheat univalents that might suggest that the P genome carries genes affecting associations between wheat chromosomes themselves. Nevertheless, pairing between P and D genomes is possible and allosyndetic pairing was clearly visible in *T. tauschii*-*A. cristatum* associations in the DDPP amphiploid [17].

The plant material developed in this work might be very useful in studying the interactions between genes included in the P genome and those controlling chromosome associations in wheat. Previously, chromosome addition lines of *A. cristatum* in wheat in the presence of the *Ph1* locus were developed for chromosomes 1P, 2P, 3P, 4P, 5P, and 6P and the ditelosomic addition line for chromosome arms 2PS, 2PL, 4PS, 5PL, 6PS, and 6PL [15,52,53]. In fact, these available wheat-*A. cristatum* addition lines allowed the location of molecular markers and genes for several important agronomic traits on specific *A. cristatum* chromosomes and chromosome arms [8,9,36,42]. However, no ditelosomic addition or substitution lines were previously obtained for the chromosome 5PS arm. Furthermore, in this work, we have developed *A. cristatum* addition lines for the 5P and 6P chromosome in wheat background in the absence of the *Ph1* locus. The availability of CS *A. cristatum* 5PS ditelosomic lines as well as the *Agropyron* introgressions in the *Ph1* mutant background provided the opportunity to not only locate genes or markers on this chromosome arm, but also to go deeper into the knowledge of chromosome associations in the wheat background.

## 4. Materials and Methods

### 4.1. Plant Material

The plant material used in this study included common wheat (*Triticum aestivum* L.) Chinese Spring (CS), CS *ph1b* mutant [54], CS/*A. cristatum* disomic addition lines for 5P and 6P chromosomes, and ditelosomic 5PL, 6PS, and 6PL addition lines [15,52,53]. The CS/*A.cristatum* addition lines for the 5P and 6P chromosomes in the CS *ph1bph1b* genetic background were selected in descendance from the crosses between the CS/*A. cristatum* disomic addition lines for 5P and 6P chromosomes with the CS *ph1b* mutant [32].

### 4.2. DNA Marker Characterization

Genomic DNA was isolated from young frozen leaf tissue using the CTAB method [55]. Samples were stored at −20 °C until PCR amplification was carried out. The concentration of each sample was estimated using a nano-drop 1000 spectrophotometer (Thermo Scientific, Waltham, MA, USA).

Four conserved orthologous set (COS) markers named COS108, COS150, COS440, and COS580 [56] were used to identify short and long arms from 5P and 6P *A. cristatum* chromosomes, respectively, included in the addition lines. These COS markers, which have been previously transferred and determined their arm locations on *A. cristatum* chromosomes [9,37], were selected for being polymorphic between *A. cristatum* and wheat CS. In detail, each COS marker was specific for each short or long 5P and 6P chromosome arm. Primer sequences for these markers and annealing temperature (Ta) were previously given [9,37]. PCR was performed with 40 ng of template DNA in a 25 μL volume reaction mixture containing 5 μL of 1× PCR buffer 0.5 pM of each primer, 1.5 mM MgCl_2_, 0.3 mM dNTPs, and 0.625U of Taq DNA polymerase (Promega, Madison, WI, USA). PCR conditions for the COS markers were as follows: 4 min at 94 °C, followed by 35 cycles of 45 s at 94 °C, 50 s at 58 °C, and 50 s at 72 °C. The PCR products were analysed in polyacrylamide gels (10% *w*/*v*, C: 2.67%) stained with ethidium bromide.

A PCR assay described by Wang et al. [57] was used to verify the presence of *Ph1*. Each 30 μL PCR contained 20 ng template DNA, 1× PCR buffer with MgCl_2_, 0.25 mM dNTP, 0.17 μM primers, and 0.02U/μL of Taq DNA polymerase (Promega, Madison, WI, USA). The reaction was first denatured (94 °C/5min), and then subjected to 35 cycles of 94 °C/60 s, 51 °C/60 s, and 72 °C/60 s, followed by a final extension (72 °C/7min). The PCR products were electrophoretically separated in a 1% agarose gel and visualized by EtBr staining.

### 4.3. Somatic Cells Analyses

Chromosome spreads were prepared from root tip cells. Seeds were germinated on wet filter paper in the dark for 3 days at 4 °C, followed by a period of 24 h at 25 °C. Root tips from germinating seeds were excised and pretreated in ice water for 24 h and then fixed in a freshly prepared ethanol–acetic acid (3:1 *v*/*v*) and stored at 4 °C for at least 1 month. The plants were grown under a greenhouse held at 26 °C during the day and 18 °C during the night at a 16 h photoperiod.

The *in situ* hybridization protocol followed that described by Cabrera et al. [58]. The probe pAs1 isolated from *Aegilops tauschii* Coss. [59] was used to determine the D genome of wheat. Genomic DNA from *A. cristatum* was used as a probe to identify P genome chromosomes. The pAs1 probe was labelled with biotin-16-dUTP (corporate Roche). Total DNA of *A. cristatum* was labelled with biotin-16-dUTP (Boehringer Mannheim Biochemicals, Germany) or digoxigenin-11-dUTP (Roche Applied Science, Indianapolis, IN, USA) using nick translation. Chromosome preparations were hybridized with an *A. cristatum* genomic DNA probe or simultaneously with both pAs1 and *A. cristatum* genomic DNA probes. The final concentration of each probe was 5 ng/μL in the hybridization mix (50% formamide, 2 × SCC, 5 ng of each digoxigenin and biotin-labelled probes, 10% dextran sulfate, 0.14 µg of yeast tRNA, 0.1 µg of sonicated salmon sperm DNA, and 5 ng of glycogen. Posthybridization washes were conducted twice at 2 × SSC (5 min each) at 37 °C plus one extra wash in 1 × SSC at room temperature (RT). Biotin- and digoxigenin-labelled probes were detected with streptavidin-Cy3 conjugates (Sigma, St. Louis, MO, USA) and antidigoxigenin FITC antibodies (Roche Diagnostics, Meylan, France), respectively. The chromosomes were counterstained with 4′,6-diamidino-2-phenylindole (DAPI) and mounted in Vectashield (Vector Laboratories, Burlingame, CA, USA). Hybridization signals were visualized using a Leica DMRB epifluorescence microscope and the images were captured with a Leica DFC 7000T camera equipped with an exposimeter spot Leica Wild MPS 52 and were processed with LEICA application suite v4.0 software (Leica, Wetzlar, Germany).

### 4.4. Meiotic Cells Analyses

GISH analysis allowed the visualization of the *A. cristatum* chromosome and their associations during meiosis in the wheat background in the presence and in the absence of the *Ph1* locus, as described previously [60]. Mature plants were used to collect spikes in meiosis, which were preserved in 100% ethanol–acetic acid 3:1 (*v*/*v*) until they were used to characterize chromosome associations. Chromosome spreads were prepared from pollen mother cells (PMCs) at meiosis. Anthers were macerated in a drop of 45% glacial acetic acid on ethanol-cleaned slides, squashed under a cover slip, and dipped in liquid nitrogen in order to fix the plant material on the slide. The cover slip was removed and the slides were air-dried and stored at 4 °C until used. Total genomic DNA from *A. cristatum* was labelled by nick translation with biotin-11-dUTP to be used as a probe for *in situ* hybridization experiments in PMCs. The conserved telomeric sequence from *A. thaliana* (AAATCCC) [61] was also labelled by nick translation with digoxigenin-11-dUTP to allow the visualization of the telomeres from wheat and *A. cristatum* chromosomes. Similar to somatic cells analysis, the final concentration of each probe was 5 ng/μL in the hybridization mix (50% formamide, 2 × SCC, 5 ng of each digoxigenin and biotin-labelled probes, 10% dextran sulfate, 0.14 µg of yeast tRNA, 0.1 µg of sonicated salmon sperm DNA, and 5 ng of glycogen). Posthybridization washes were equivalent to the ones performed for somatic cells analysis. Biotin-labelled and digoxigenin-labelled probes were detected with a streptavidin-Cy3 conjugate and antidigoxigenin-FITC, respectively. Chromosomes were counterstained with DAPI (4′,6-diamidino-2-phenylindole) and mounted in Vectashield (Vector Laboratories, Burlingame, CA, USA). Hybridization results were imagined using a Nikon Eclipse 80i epifluorescence microscope. Images were taken with a Nikon CCD camera using the Nikon 3.0 software (Nikon Instruments Europe BV, Amstelveen, The Netherlands) and processed with Photoshop 11.0.2 software for adjustment of brightness and contrast (Adobe Systems Inc., San Jose, CA, USA).

## 5. Conclusions

The development of *A. cristatum* 5P and 6P chromosomes in wheat in the *Ph1* mutant background revealed that chromosome associations between *A. cristatum* and wheat chromosomes are not performed during meiosis, even in the absence of the *Ph1* locus, although the *A. cristatum* genome might have a putative effect on homologous chromosome associations in wheat itself.

## Figures and Tables

**Table 1:** Analysis of metaphase I cells carrying unassociated (univalent) wheat chromosomes in the presence of 5P and 6P *A. cristatum* chromosomes.

Number of Metaphase I Cells Analysed	Number (and Percentage of Cells Carrying One, Two, or Three Wheat Chromosomes as Univalents				Total Number (and Percentage) of Cells Carrying Wheat Univalents
		I	II	III	
Chromosome 5P					
Monosomic CS+5P *Ph1Ph1*	100	5 (5.0%)	11 (11.0%)	2 (2.0%)	18 (18.0%)
Disomic CS+5P *Ph1Ph1*	132	14 (10.6%)	18 (13.7%)	3 (2.3%)	35 (26.5%)
Monosomic CS+5P *ph1ph1*	129	8 (6.2%)	28 (21.7%)	4 (3.1%)	40 (31.0%)
Disomic CS+5P *ph1ph1*	121	16 (13.2%)	21 (17.3%)	3 (2.5%)	40 (33.0%)
Chromosome 6P					
Monosomic CS+6P *ph1ph1*	116	2 (1.7%)	15 (13.0%)	14 (12.1%)	31 (26.7%)
Disomic CS+6P *Ph1Ph1*	117	10 (8.6%)	33 (28.2%)	3 (2.6%)	46 (39.3%)
